# Draft Genome Sequences of Longimonas halophila KCTC 42399 and Longibacter salinarum KCTC 52045

**DOI:** 10.1128/MRA.01238-19

**Published:** 2019-11-14

**Authors:** Kok Jun Liew, Seng Chong Teo, Mohd Shahir Shamsir, Kian Mau Goh

**Affiliations:** a Faculty of Science, Universiti Teknologi Malaysia, Skudai, Johor, Malaysia; University of Maryland School of Medicine

## Abstract

Longimonas halophila and Longibacter salinarum are type strains of underexplored genera affiliated with *Salisaetaceae*. Herein, we report the draft genome sequences of two strains of these bacteria, L. halophila KCTC 42399 and L. salinarum KCTC 52045, with the intent of broadening knowledge of this family. Genome annotation and gene mining revealed that both bacteria exhibit amylolytic abilities.

## ANNOUNCEMENT

The order *Rhodothermales* consists of the families *Rhodothermaceae*, *Salisaetaceae*, *Salinibacteraceae*, and *Rubricoccaceae. Rhodothermaceae* was proposed 30 years ago, while the *Salisaetaceae* family was suggested in early 2019 (1). The family *Salisaetaceae* consists of four known genera, Longimonas, Longibacter, Natronotalea, and Salisaeta. These bacteria are Gram negative, aerobic, rod shaped, orange or red pigmented, nonmotile, and heterotrophic and require NaCl for growth (2–5). All related type strains for the genera stated above have not been genomically analyzed, except for Salisaeta longa DSM 21114 (GenBank accession number ATTH00000000) (5). Here, we present the genome sequences and analyses of Longimonas halophila and Longibacter salinarum.

*L. halophila* and *L. salinarum* were obtained from the Korean Collection for Type Cultures (KCTC; deposition numbers KCTC 42399 and KCTC 52045, respectively). Both bacteria were initially isolated from a marine solar saltern located in China (2, 3). The bacteria were grown on Marine agar 2216 (BD Diagnostic Systems, Sparks, MD) with the addition of 7% (wt/vol) NaCl at 37°C (pH 7.5) for 5 days. Genomic DNA was extracted from the bacteria using the Quick-DNA Miniprep Plus kit (Zymo Research, Irvine, CA).

The extracted genomes were constructed into paired-end 300-bp libraries using a Nextera sample prep kit and sequenced on a MiSeq sequencer with v2 chemistry (Illumina, San Diego, CA). The reads were quality checked and the adapters trimmed using Trimmomatic v0.36 (6). *De novo* assembly of the genome was performed using SPAdes v3.10.1 (7). The genomes were analyzed and annotated via the NCBI Prokaryotic Genome Annotation Pipeline (PGAP) v2.10 (8). The annotated genes were then assigned using eggNOG 5 (evolutionary genealogy of genes: Non-supervised Orthologous Groups) (9). Carbohydrate-active enzymes (CAZymes) present in the genome were gene mined using Carbohydrate-active enzyme Annotation v2 (dbCAN2) (10). Default parameters were used for all software.

For *L. halophila*, the sequencer generated 1,369,214,354 bases from 2,309,577 paired-end reads. Upon removing adapters and low-quality data, the reads were assembled into 35 contigs with a coverage of 288×. This bacterium harbors a genome of 3,729,970 bp, with an *N*_50_ value of 249,688 bp. The G+C content of the genome was around 60.5%. A total of 2,997 genes were predicted. Of these, around 87% (2,604 genes) are associated with Cluster of Orthologous Groups (COG) functions. *L. halophila* encodes 121 CAZymes, including 31 glycoside hydrolases (GHs), 55 glycosyltransferases (GTs), 5 polysaccharide lyases (PLs), 26 carbohydrate esterases (CEs), and 4 auxiliary activity enzymes (AAs). *L. halophila* tested positive on starch due to the presence of five various α-amylases and two pullulanases that act on α-1,4- and α-1,6-glycosidic linkages, respectively.

Approximately 1.03 billion bases in 1,738,070 paired-end reads were obtained from *L. salinarum* genome sequencing. After removing adapters and low-quality reads, the cleaned-up reads were assembled into 22 contigs with a coverage of 179×. The draft genome is 4,406,485 bp, with an *N*_50_ value of 433,789 bp. The G+C content of the genome was around 59.3%. A total of 3,486 genes were predicted, with 85% (2,979 genes) of the total genes linked to COG functions. The total number of CAZymes encoded was 140 (36 GHs, 61 GTs, 8 PLs, 31 CEs, and 4 AAs).

Collectively, genome sequencing and gene mining suggested that both strains exhibit amylolytic ability against starch. This article presents the genome sequences of the two bacteria, which contribute to a further understanding of the genera and their family.

### Data availability.

The whole-genome shotgun projects of *L. halophila* and *L. salinarum* have been deposited in GenBank with accession numbers PDEP00000000 and PDEQ00000000, respectively. The raw sequencing reads have been deposited in the NCBI Sequence Read Archive (SRA) with accession numbers SRR10190493 for *L. halophila* and SRR10190806 for *L. salinarum*.
